# Impact of a care bundle on short peripheral catheter-associated complications in a resource-limited neonatal unit

**DOI:** 10.1177/11297298241278394

**Published:** 2024-09-19

**Authors:** Angela Dramowski, Louisa Erasmus, Aaqilah Fataar, Mark F Cotton, Andrew C Whitelaw, Susan Coffin, Adrie Bekker

**Affiliations:** 1 Department of Paediatrics and Child Health, Faculty of Medicine and Health Sciences, Stellenbosch University, Cape Town, South Africa; 2 Division of Medical Microbiology, Department of Pathology, Faculty of Medicine and Health Sciences, Stellenbosch University and National Health Laboratory Service, Tygerberg Hospital, Cape Town, South Africa; 3 Division of Infectious Diseases, Children’s Hospital of Philadelphia and Department of Paediatrics, University of Pennsylvania Perelman School of Medicine, Philadelphia, PA, USA

**Keywords:** Peripheral intravenous catheter, dwell time, infection prevention, neonate, healthcare-associated infection, device-associated infection, care bundle

## Abstract

**Background:**

Short peripheral catheter (SPC)-associated complications occur frequently in hospitalised neonates. Few studies have reported the use of SPC care bundles in resource-limited neonatal units.

**Objective:**

To evaluate the impact of a SPC care bundle on SPC associated complications (infiltration, dislodgement, phlebitis) and catheter dwell time.

**Methods:**

We conducted a quasi-experimental study comparing neonatal SPC complications during a 2-month baseline and a 2-month intervention period, where a SPC care bundle was introduced including hand hygiene, insertion site antisepsis, nurse assistance during cannulation, IV insertion carts and IV securement dressings.

**Results:**

A total of 459 SPC days were observed in 223 neonates: 111 pre-intervention and 112 post-intervention (after SPC bundle implementation). Most neonates were preterm (208, 93.3%) with very or extremely low birth weight (133, 59.6%). SPC care bundle compliance was 43.8% for five bundle elements and 83.9% for four bundle elements. Most SPCs had unplanned removal within 48 h of insertion owing to infiltration or dislodgement (89/111 pre-intervention (80.2%) vs 90/112 post-intervention (80.4%); 0.974). No phlebitis was documented. The mean SPC dwell time was unchanged following bundle implementation (32.9 vs 34.2 h; *p* = 0.376).

**Conclusions:**

Infiltration and dislodgement occurred frequently necessitating replacement of four of every five SPCs. Despite moderate compliance with the SPC care bundle, the high rates of unplanned SPC removal and short duration of catheter dwell time were unchanged.

**Contribution:**

The SPC care bundle did not improve catheter dwell time; further research is needed to identify strategies to reduce unplanned SPC removal and extend catheter dwell time in hospitalised neonates.

## Introduction

Short peripheral intravenous catheters (SPCs) are the most frequently used invasive medical devices and an essential part of medical care for most low birthweight infants admitted to the neonatal intensive care unit (NICU) or neonatal wards.^1^ The main indications for SPC placement include drug administration, infusion of fluids compatible with the peripheral route, partial parenteral nutrition and blood products.^2^

Despite the availability and use of infusion therapy standards of practice (INS 2021),^3^ SPC complications such as pain, infiltration, accidental dislodgement, catheter occlusion and thrombophlebitis occur frequently.^4–6^ Loss of intravenous (IV) access usually necessitates insertion of a new SPC, resulting in treatment delays, increased costs and repeated skin barrier disruptions, with the latter increasing risks of healthcare-associated infection (HAI).^7^ Healthcare-associated bloodstream infections (BSI) are among the most severe SPC-associated complications and the most frequent HAI type encountered in hospitalised neonates.^8,9^

Care bundles are frequently used infection prevention tools, which apply several evidence-based interventions in combination to reduce device-associated infections that is, for central lines or urinary catheters.^7,10–13^ Care bundles have also been developed to prevent SPC-associated BSI, incorporating both insertion and maintenance bundle elements.^7,11,14,15^ Elements include hand hygiene prior to SPC placement and manipulation, daily review of SPC dressings and insertion site, and prompt removal of unutilised, infiltrated or infected SPCs.^14,15^ The heterogeneity of bundle components complicates direct assessment of how each bundle element influences efficacy.^7^ In a systematic review of SPC bundle effectiveness, six studies reported reduction in BSI and phlebitis rates, and another observed no change in BSI rates but an increase in phlebitis rates.^7^

Although meta-analysis confirmed the effectiveness of central line-associated BSI care bundles in neonates,^16^ data on neonatal SPC care bundles is limited and mostly generated from high-income countries and neonatal intensive care unit (NICU) settings.^17–21^ In an observational study of SPC use in hospitalised neonates and children, the mean SPC dwell time was 42 h, with phlebitis present in 6% at SPC removal; neonates were five times more likely to develop phlebitis than children.^17^ In another multi-site study, a third (35%) of SPCs required removal due to infiltration or occlusion. Neonates also had significantly shorter SPC dwell time than children (4 vs 5 days).^2^ In a prospective observational study of SPC-related complications at 2 Dutch neonatal ICUs, extravasation was the leading reason for non-elective SPC removal.^6^ In a randomised controlled trial, routine SPC replacement in neonates between 72 and 96 h of use did not reduce the extravasation injury rate, phlebitis or spontaneous dislodgement.^18^ Other factors affecting infiltration and extravasation risk in neonates include administration of hypertonic solutions (e.g. glucose at concentrations exceeding 10%) and vasoactive/irritant medicines (e.g. pressors, vancomycin).^19,20^

There is extremely limited data evaluating the implementation of SPC care bundles in neonates from non-ICU and resource-limited settings. In this quasi-experimental, pre-post intervention study, we evaluated the impact of a SPC insertion care bundle on catheter dwell time at a large South African neonatal unit.

## Methods

### Study design and population

We conducted a quasi-experimental, pre-post intervention study to evaluate the impact of a SPC care bundle on catheter dwell time in two 30-bed acute care wards at the Tygerberg Hospital neonatal unit, Cape Town, South Africa. We enrolled neonates requiring SPC insertion during weekday working hours over a 2-month baseline period and a 2-month intervention period (September-December 2020). Enrolled neonates were observed for SPC dwell time and complications (infiltration, dislodgement and phlebitis) until the catheter was removed or dislodged, up to a maximum of 7 days. The Stellenbosch University Health Research Ethics Committee and the Tygerberg Hospital management reviewed and approved the study protocol (N18/07/068).

### Study setting

Tygerberg Hospital is a 1384-bed public teaching hospital. Its busy obstetric-neonatal service has 8000 high-risk deliveries (37% low birth weight rate) and 3000 neonatal admissions annually, with neonatal ward bed occupancy rates fluctuating from 90% to 105%. The neonatal unit, the second largest in South Africa, has 132 beds including 12 NICU beds, three 30-bed high-dependency wards and a kangaroo mother care ward. The neonatal unit provides medical and surgical care for sick, preterm (<37 weeks’ gestation) and/or low-birthweight (<2500 g) inborn and outborn neonates from surrounding district hospitals and midwife obstetric units. Prematurity, perinatal asphyxia and infection are the most common indications for admission. Given the limited availability of NICU beds, non-invasive ventilation and surfactant therapy is administered in the three acute care neonatal wards.

### SPC standard of care

Neonatal SPCs (Jelco^®^ IV catheters, 24G) are inserted at ward admission by doctors including paediatric residents, medical officers and interns, usually without nurse assistance. SPCs are sited for administration of IV fluids, blood products and IV medication. Central lines are used only if absolutely indicated that is, no peripheral access, need for parenteral nutrition, inotropes or >10% dextrose-containing solutions. All solutions, infusions and drugs administered through a SPC are routinely checked for compatibility with the peripheral administration route. Parenteral nutrition is only administered through central lines in the neonatal unit. The attending clinician selects the neonate’s peripheral vein deemed most suitable for cannulation that is, a straight vein, of sufficient diameter that does not cross a joint. Hand hygiene prior to SPC insertion is performed using 70% isopropyl alcohol-based handrub located at every neonate’s bedside. Use of disposable gloves (or sterile gloves when available) is encouraged and recommended in the standard operating procedures (SOP) for SPC insertion. Pre-insertion skin antisepsis is performed using small pre-packed alcohol wipes. Prior to bundle implementation, there was no assistant for IV insertion, no IV procedure cart and no access to specialised IV securement dressings.

### SPC care bundle

The SPC bundle intervention was designed in consultation with neonatal unit clinicians and the infection prevention nurse practitioner following review of published literature. The SPC insertion care bundle elements included (1) hand hygiene using alcohol handrub, (2) enhanced insertion site skin antisepsis, (3) having nurse assistance during cannulation, (4) use of an IV insertion cart and (5) enhanced neonatal IV securement dressings. Enhanced skin antisepsis was performed using sterile cotton wool swabs soaked in 0.5% chlorhexidine gluconate in 70% alcohol to clean the skin using friction for 20–30 s, allowing time for the neonate’s skin to dry prior to SPC insertion. A nurse assistant during SPC insertion, a dedicated IV insertion cart and transparent IV securement dressings (Tegaderm IV advanced neonatal 1680 – 3M™) were introduced as part of the care bundle. A research nurse observed staff compliance with the standard of care SPC insertion practices (during the baseline phase) and staff compliance with the SPC bundle (during the intervention phase).

### Training of neonatal unit staff

**Figure 1.**
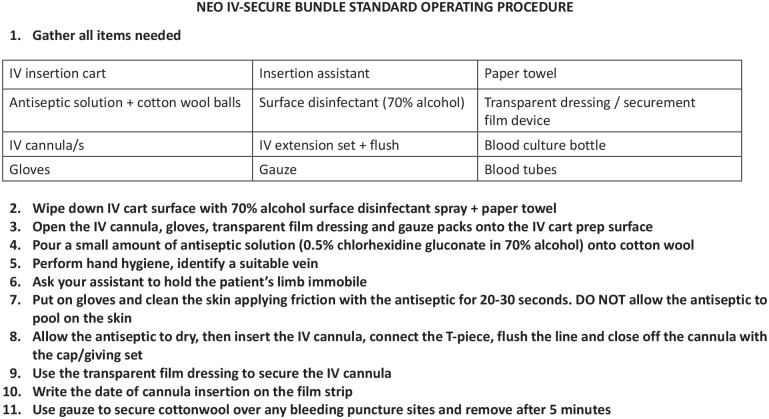
Standard operating procedure for the short peripheral cannula insertion care bundle.

Medical staff in the neonatal unit (paediatric residents, medical officers and interns) received face-to-face training in the wards from the study investigator and research nurse. Training included a careful step-by-step review of the new Neo IV-SECURE SPC insertion SOP, showing staff where to find each consumable needed for the procedure in the SPC insertion cart for example, surface disinfectant, alcohol hand rub, sterile cotton swabs, antiseptic, securement devices. A laminated copy of the SPC insertion SOP was attached to the IV cart for easy reference while performing cannulation (Figure 1). Demonstrations of how to perform enhanced skin antisepsis and how to apply the IV securement dressing were conducted in each ward.

### Data points and outcomes of interest

For each enrolled neonate undergoing cannulation, demographics (sex, birth weight, gestational age, admission diagnosis), date and time, seniority of medical staff member, number of cannulation attempts and reason for requiring a SPC was recorded on a case report from. Neonates were visited daily on weekdays by the study nurse to review the SPC status (intact, extravasated, phlebitis, unplanned removal i.e. dislodged/occluded or planned removal i.e. IV medication/infusion therapy completed). Data on SPC status or complications that occurred afterhours (nights and weekends) was retrospectively collected and documented in the case report form on the next working day. The primary outcome of interest was SPC dwell time, with secondary outcomes including infiltration, dislodgement and phlebitis. Neonates’ SPCs were observed for a maximum of 7 days post-insertion, or until SPC removal, whether planned or unplanned. Compliance with the SPC insertion care bundle was measured during the bundle implementation phase by adding number of instances where staff were compliant with each bundle element, divided by the total opportunities to implement each element multiplied by 100 to calculate percentage compliance.

### Statistical analysis

Comparison of neonatal demographic characteristics before-and-after bundle implementation was performed, using the Student *t*-test for continuous variables and Pearson’s χ^2^ test for categorical variables. Catheter dwell time before-and-after bundle implementation was compared using the Student *t*-test. The SPC complication rates (infiltration, dislodgement and phlebitis) were compared using Poisson regression. Sub-analyses were conducted to compare the median number of SPC insertion attempts and median catheter dwell time in the presence and absence of nurse assistance during cannulation. For all statistical tests performed, a *p*-value <0.05 was considered significant. All the statistical analyses were performed using STATA 17.0 (College Station, Texas 77845 USA).

## Results

### Neonatal characteristics

**Figure 2.**
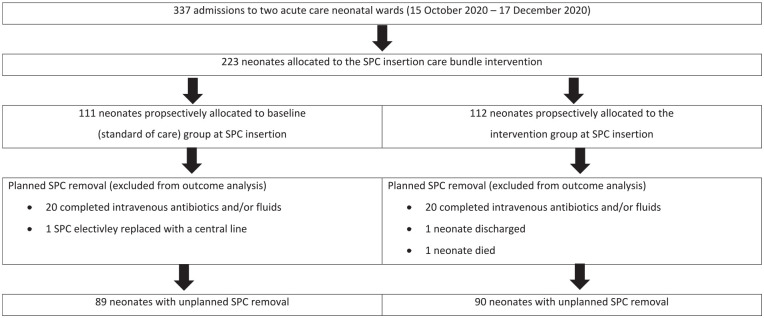
Allocation to the short peripheral catheter insertion care bundle intervention (*n* = 223). SPC: short peripheral catheter.

A total of 459 SPC catheter days were observed in 223 individual neonates: 111 pre-intervention and 112 post-intervention that is, after SPC bundle implementation (Figure 2). Most neonates (208; 93.3%) were preterm: early preterm (<32 weeks gestation; 126; 56.5%), late preterm (32–36 weeks gestation; 82; 36.8%) and of extremely low (<1000 g) or very low birth weight (1000–1499 g; 133; 59.6%). Hyaline membrane disease was the most common admission diagnosis in both time periods. Requirement for IV fluids (58.7%) and IV antibiotic administration (37.7%) were the most frequent indications for SPC insertion. Few neonates (7/223; 3.1%) received hypertonic fluids, irritant or vasoactive medications through their SPC. There were no significant differences in sex distribution, birth weight, gestational age, day of life, site of SPC insertion or seniority of physician inserting the SPC between the baseline and intervention periods (Table 1).

**Table 1.** Characteristics of neonates undergoing short peripheral catheter insertion (*N* = 223).

Variable	Total patients *n* = 223	Baseline *n* = 111	Intervention *n* = 112	*p*-Value
Gender (male)	93 (41.7)	45 (40.5)	48 (42.9)	0.726
Birth weight (g)*	1315 (1015–1765)	1405 (970–1810)	1288 (1025–1750)	0.693
Gestational age (weeks)*	31.0 (28–33)	31.2 (29–33)	30.6 (28–33)	0.173
Admission diagnosis				
Hyaline membrane disease	147 (65.9)	67 (60.4)	80 (71.4)	–
Neonatal jaundice	59 (26.5)	43 (38.7)	16 (14.3)	
Proven or presumed infection	20 (9.0)	7 (6.3)	13 (11.6)	
Necrotising enterocolitis	15 (6.7)	11 (9.9)	4 (3.6)	
Congenital syphilis	10 (4.5)	6 (5.4)	4 (3.6)	
Neonatal encephalopathy	5 (2.2)	3 (2.7)	2 (1.8)	
Day of life at SPC insertion*	3 (1–6)	3 (1–5)	3 (1–6)	0.431
Primary reason for SPC insertion				
Intravenous fluids	131 (58.7)	65 (58.6)	66 (58.9)	0.533
Antibiotic administration	84 (37.7)	40 (36.0)	44 (39.3)	
Intravenous medication	4 (1.8)	3 (2.7)	1 (0.9)	
Blood transfusion	4 (1.8)	3 (2.7)	1 (0.9)	
Fluids and medications given via SPC				
Maintenance fluid (10% dextrose)	128 (57.4)	65 (58.6)	63 (56.3)	–
Hypertonic (>10% glucose)	3 (1.4)	0 (0)	3 (2.7)	
Irritant (vancomycin)	3 (1.4)	3 (2.7)	0 (0)	
Vasoactive (pressors)	1 (0.4)	1 (0.9)	0 (0)	
Site of SPC placement				
Hand	75 (33.6)	34 (30.6)	41 (36.6)	0.678
Antecubital fossa	60 (26.9)	33 (29.7)	27 (24.1)	
Foot	60 (26.9)	31 (27.9)	29 (25.9)	
Head	24 (10.7)	12 (10.8)	12 (10.7)	
Leg	4 (1.8)	1 (0.9)	3 (2.7)	
Seniority of physician siting the SPC				
Paediatric resident	108 (48.4)	54 (48.7)	54 (48.2)	0.936
Medical officer	83 (37.2)	42 (37.8)	41 (36.6)	
Intern	32 (14.4)	15 (13.5)	17 (15.2)	

SPC: short peripheral catheter.

* Values expressed as median (interquartile range), all others as number (percentage).

### Care bundle compliance

The median number of SPC insertion attempts required to successfully complete cannulation was 1 (IQR 1–2, maximum 11 attempts); 30/223 (13.4%) neonates had >3 SPC insertion attempts (Table 2). A nurse assistant was infrequently used for SPC insertion at baseline (25; 22.5%) but was more frequently available to assist with SPC insertion during the intervention period (58; 51.8%); *p* < 0.001 (Table 2). Staff compliance with the SPC care bundle was 43.8% for all 5 bundle elements, and 83.9% for 4/5 elements (if the nurse assistant element was excluded from the bundle compliance analysis; Table 2). Compliance was highest with the hand hygiene element (100%), use of the IV securement dressing (99.1%) and application of CHG skin antisepsis (96.4%). A nurse assistant was available for only 51.8% of SPC insertions. There was a significantly higher median number of SPC insertion attempts when nurse assistants were not present at cannulation (2 (IQR 1–3) vs 1 (IQR 1–2); *p* = 0.003).

**Table 2.** Short peripheral catheter insertion practices and outcomes prior to and after bundle implementation.

Short peripheral catheter insertion practices and outcomes	Total patients *n* = 223	Baseline *n* = 111	Intervention *n* = 112	*p*-Value
SPC preparation surface				
Incubator or bedside cabinet top	126 (56.5)	111 (100)	5 (4.5)	<0.001
Dedicated IV cart	97 (43.5)	0 (0)	107 (95.5)	
Hand hygiene compliance before SPC insertion	207 (92.8)	95 (85.6)	112 (100)	<0.001
Antiseptic prior to SPC insertion				
Alcohol-impregnated swab	114 (51.1)	110 (99.1)	4 (3.6)	<0.001
0.5% CHG in 70% alcohol solution	109 (48.9)	1 (0.9)	108 (96.4)	
Method of SPC securement				
Transparent adhesive film	112 (50.2)	111 (100)	1 (0.9)	<0.001
Proprietary IV securement film	111 (49.8)	0 (0)	111 (99.1)	
Assistant used for SPC insertion	83 (37.2)	25 (22.5)	58 (51.8)	<0.001
Bundle compliance				
Four elements (excluding assistant)	–	–	94 (83.9)	–
All five elements	–	–	49 (43.8)	
Number of SPC insertion attempts, median (IQR)	1 (1–2)	1 (1–3)	1 (1–2)	0.057
Removal of SPC				
Planned	44 (19.7)	22 (19.8)	22 (19.6)	0.974
Unplanned	179 (80.3)	89 (80.2)	90 (80.4)	
Reason for unplanned SPC removal				
Total	179 (100)	89 (100)	90 (100)	0.539
Infiltration	166 (92.7)	85 (95.5)	81 (90)	0.253
Dislodgement	13 (7.3)	4 (4.5)	9 (10)	–
Phlebitis	0 (0)	0 (0)	0 (0)	
	Baseline (*n* = 89)	Intervention (*n* = 90)	Difference (95% CI)	*p*-Value
Unplanned SPC removal event rate* (*n* = 179)	*N* (event rate)	*N* (event rate)		
Infiltration	85 (51.2)	81 (47.6)	3.6 (−1.1 to 18.6)	0.644
Dislodgement	4 (2.4)	9 (5.3)	−2.9 (−7.1 to 1.3)	0.193
Phlebitis	0 (0)	0 (0)	–	–
SPC dwell time in hours, mean (SD)	32.9 (22.2)	34.2 (32.2)	1.3 (−6.9 to 9.5)	0.376

IV: intravenous; CHG: chlorhexidine gluconate; SPC: short peripheral catheter; IQR: interquartile range; CI: confidence interval; SD: standard deviation.

All values expressed as *n* (%), unless otherwise specified.

* Neonates with planned SPC removal were excluded from this analysis (e.g. completed antibiotics or fluids, discharged, transferred, died or SPC replaced with a central line); rate expressed per 100 SPC days.

### Outcome measures

Most SPCs had unplanned removal owing to tissue infiltration or dislodgement (89/111 at baseline (80.2%) vs 90/112 during the intervention period (80.4%); 0.974). The mean SPC dwell time was unchanged following bundle implementation (32.9 vs 34.2 h; *p* = 0.376; Table 2). There was no difference in the median SPC dwell time by presence of nurse assistance during cannulation (*p* = 0.1). There was no difference in the rate of infiltration or dislodgement for neonates with unplanned SPC removal comparing the pre-intervention to the post-intervention period (Table 2). No phlebitis was documented.

## Discussion

SPC insertion is a frequently performed invasive procedure in neonates and is commonly associated with adverse events. Despite availability of evidence-based guidelines,^3^ and reports of successful central venous access and SPC care bundles, limited data is available on SPC quality improvement in resource-limited neonatal care settings.^7,22–27^ In this quasi-experimental, pre-post intervention study, we evaluated the impact of a SPC care bundle on catheter dwell time at a large South African neonatal unit.

### Neonatal and SPC characteristics

Most neonates who required SPC insertion in this cohort were preterm, of low birth weight and required a SPC for intravenous fluids, antibiotics or both. In most neonates, SPCs were inserted by experienced doctors (residents and medical officers), with infrequent use of a nurse assistant during cannulation at study baseline. Much higher rates of unplanned SPC removal were observed in this neonatal cohort (80%) compared to other prospective neonatal (56%)^6^ and paediatric (50%)^28^ studies. In alignment with previous reports, the leading reason for unplanned SPC removal was infiltration and less commonly, dislodgement. Other studies have demonstrated rates of extravasation up to 70% in neonates.^6,20,21,29^ This population is at increased risk of complications compared to adults owing to physical factors like smaller, more fragile veins, sensitive skin and flexible subcutaneous tissue, leading to difficulty in inserting and securing SPCs.^29^

### Care bundle compliance and study outcome

Although moderate SPC bundle compliance was achieved, the mean SPC dwell time and rate of unplanned SPC removal was unchanged. Very short mean SPC dwell times were observed in our study (32.9 h baseline vs 34.2 h post-bundle implementation; *p* = 0.376). This is substantially shorter than the dwell time reported in Dutch neonatal units of 47 h (SD: 43 h)^6^ but similar to that reported from an Indian neonatal unit at 32 h (SD: 16 h).^30^ Another study of neonates in Ethiopian public hospitals demonstrated that 83% of SPCs remained in situ for <48 h after insertion.^31^

Very high compliance rates were achieved for four bundle elements during the intervention: hand hygiene and skin antisepsis prior to SPC insertion, use of the IV insertion cart and use of IV securement dressings. Compliance was lowest for nurse assistance during SPC insertion, possibly because the dedicated research nurse covered multiple neonatal wards and the ward nurses were frequently busy with other tasks. High patient to staff ratios in resource-limited neonatal wards may present challenges to SPC bundle compliance.

No reduction in SPC infiltration rates was achieved in this study following implementation of the bundle. This contrasts with two paediatric studies that reported small reductions in SPC infiltration rates. One Thai study achieved a 3.5% reduction in SPC infiltration rates following an infiltration-focused insertion and maintenance bundle.^32^ Additional bundle elements in that study included assessment of vein condition prior to SPC insertion, appropriate site selection and assessment of SPC status at each shift. A Korean paediatric unit demonstrated a 4.8% reduction in SPC infiltration rates following implementation of a maintenance bundle with daily review of line necessity, hand hygiene, site assessment and dressing checks.^33^ No confirmed SPC-related infections, phlebitis or associated bloodstream infections were observed in our study, probably linked to the very short dwell time.

Although no change in the SPC dwell time was achieved after SPC bundle introduction, the development of guidelines for SPC insertion was important to standardise practice and training of new neonatal ward staff for this common medical procedure. In the two neonatal SPC bundle studies described above from resource-limited settings, only modest reductions in SPC infiltration rates were achieved (3%–5%). The exact reason for the lack of impact of our SPC bundle on dwell time and infiltration rates is unclear, as moderate bundle compliance rates were observed. It is possible that the lack of effect on SPC infiltration may be due to the unique characteristics of the study population with poor soft tissue integrity in preterm neonates. In addition, the skin antisepsis bundle element targets prevention of SPC-associated skin and soft tissue infection and would not contribute to reduction in SPC infiltration rates. The sub-set analyses for the presence of a nurse assistant and the number of IV insertion attempts and dwell time, also showed no impact. Therefore, improving these two elements (glove wearing and nurse assistance), seem unlikely to improve SPC dwell time in our setting.

One potentially very important factor influencing SPC dwell time is SPC maintenance care post-insertion during accessing or manipulation. Future neonatal SPC care bundles aiming to prolong dwell time should include elements focused on IV line maintenance for example, regular inspection for loose dressings, early signs of tissue infiltration and best practices for manipulating/accessing the line. Other interventions that may improve SPC dwell time and reduce dislodgement/infiltration are use of smaller gauge catheters (26G), cannulas with stabilisation wings and use of sterile cyanoacrylate glue to stabilise the device.^3^ Use of octyl-butyl cyanoacrylate adhesive glue as part of neonatal SPC insertion practice was recently shown to reduce phlebitis by 77% and extend dwell time by 20%.^34^ In addition, continuous infusion site monitoring via sensor technology has shown promise in earlier detection of SPC infiltration and extravasation compared to intermittent observation alone.^35^ The ideal combination of bundle elements for SPC insertion and maintenance in resource-limited neonatal units is however, unclear at present.

Reducing the number of attempts required to insert a SPC may limit the number of painful procedures and skin breaches in neonates. Difficulty in inserting a SPC on the first attempt is common, especially in preterm neonates. This study almost achieved a significant reduction in the number of SPC insertion attempts with a median of 1 attempt required (range: 1–11 attempts), similar to findings in Dutch and British studies that reported a median of 2 (range: 1–10) SPC insertion attempts in neonates.^6,20^ The low number of attempts required to insert a SPC in our setting may be attributable to the high level of experience among paediatric residents and medical officers in our setting, who performed the majority (85%) of SPC insertions. This is in contrast Indian neonatal units where only 35% of SPCs are placed by senior medical practitioners.^30^ An important additional consideration for future studies is to compare the cost, utility, dwell times and complication rates associated with standard SPC versus those with extended dwell times such as epicutaneo-caval catheter (ECC), and umbilical venous catheters (UVCs). In a large single centre cohort study describing outcomes of 23,858 vascular access devices, central venous devices were associated with lower complication rates than SPCs.^5^

Limitations of this study are the small sample size and implementation in a single, tertiary care neonatal unit. In addition, the presence of the research nurse assistant during cannulation in the intervention period may have influenced doctors’ compliance with hand hygiene and skin antisepsis recommendations. We did not record compliance with the use of gloves during SPC insertion, although this practice was recommended in the IV insertion SOP. We also did not include or evaluate SPC maintenance strategies such as no touch techniques for SPC access or regular surveillance of the SPC placement site. Despite these limitations, this study heightened awareness of the SPC insertion bundle elements and resulted in improved compliance, although no difference in complication rates or dwell time was achieved.

## Conclusion

Unplanned SPC removal owing to tissue infiltration or dislodgement occurred frequently, necessitating replacement of four in every five neonatal SPCs. Despite satisfactory compliance with the SPC insertion care bundle, there was no change in the rate of unplanned SPC removal or mean SPC dwell time. Current evidence for use of SPC care bundles to reduce adverse patient outcomes is promising, but not robust in resource-limited settings. Further research is needed to identify which strategies and devices are most effective at extending SPC dwell time in neonates.
